# Pharmacokinetics and pharmacogenomics of ribociclib in black patients with metastatic breast cancer the LEANORA study

**DOI:** 10.1038/s41523-024-00692-w

**Published:** 2024-09-30

**Authors:** Ilana Schlam, D. Max Smith, Cody Peer, Tristan Sissung, Keith T. Schmidt, Ming Tan, Ami Chitalia, Nanette H. Bishopric, Seth Steinberg, Hyoyoung Choo-Wosoba, Giulia Napoli, Christopher Gallagher, Nadia Ashai, Kristen Whitaker, Candace Mainor, Shruti Tiwari, Nicole Swanson, Stacy Malloy, Claudine Isaacs, William Douglas Figg, Sandra M. Swain

**Affiliations:** 1 Division of Hematology and Oncology, Tufts Medical Center, Boston, MA USA; 2 Tufts University, Boston, MA USA; 3 Department of Oncology, Georgetown University Medical Center, Washington, DC USA; 4 MedStar Health, Columbia, MD USA; 5 Georgetown Lombardi Comprehensive Cancer Center, Washington, DC USA; 6 Clinical Pharmacology Program, National Cancer Institute, Bethesda, MD USA; 7 Molecular Pharmacology Section, Genitourinary Malignancies Branch, Center for Cancer Research, National Cancer Institute, National Institutes of Health, Bethesda, MD USA; 8 Department of Biostatistics, Bioinformatics and Biomathematics, Georgetown University Medical Center, Washington, DC USA; 9 Hematology-Oncology Department, MedStar Washington Hospital Center, Washington, DC USA; 10 Office of Collaborative Biostatistics, Center for Cancer Research, National Cancer Institute, National Institutes of Health, Bethesda, MD USA; 11 Virginia Cancer Specialists, Fairfax, VA USA

**Keywords:** Breast cancer, Genetics research, Breast cancer

## Abstract

Underrepresented populations’ participation in clinical trials remains limited, and the potential impact of genomic variants on drug metabolism remains elusive. This study aimed to assess the pharmacokinetics (PK) and pharmacogenomics (PGx) of ribociclib in self-identified Black women with hormone receptor-positive (HR+)/human epidermal growth factor receptor 2-negative (HER2) advanced breast cancer. LEANORA (NCT04657679) was a prospective, observational, multicenter cohort study involving 14 Black women. PK and PGx were evaluated using tandem mass spectrometry and PharmacoScan™ microarray (including CYP3A5*3, *6, and *7). CYP3A5 phenotypes varied among participants: 7 poor metabolizers (PM), 6 intermediate metabolizers (IM), and one normal metabolizer (NM). The area under the curve did not significantly differ between PMs (39,230 h*ng/mL) and IM/NMs (43,546 h*ng/mL; *p* = 0.38). The incidence of adverse events (AEs) was also similar. We found no association between *CYP3A5* genotype and ribociclib exposure. Continued efforts are needed to include diverse populations in clinical trials to ensure equitable treatment outcomes.

## Introduction

Breast cancers can be subclassified based on receptors on tumor cells; these play a critical role in tumor biology and determining the optimal treatment approach for each patient^1^. Hormone (estrogen and/or progesterone) receptor-positive (HR+) and human epidermal growth factor receptor 2 negative (HER2-) breast cancer accounts for approximately 70% of all breast cancers^1^. The cornerstone of treatment for patients with endocrine-sensitive advanced HR+/HER2− breast cancer is endocrine therapy (ET), which consists of selective estrogen receptor modulators (SERM: tamoxifen), aromatase inhibitors (AIs: letrozole, anastrozole, exemestane) or selective estrogen receptor degraders (SERDs: fulvestrant)^2,3^. However, patients often develop resistance to these treatments, and progression of disease. Novel agents have been added to ET with the goal of improving patient outcomes.

When added to ET, the cyclin-dependent kinase 4 and 6 inhibitors (CDK4/6i) palbociclib, ribociclib, and abemaciclib improve progression-free survival (PFS)^4–10^. In the first-line setting, ribociclib also led to a statistically significant improvement in overall survival (OS), when added to an AI^11^. These oral agents have had a profound impact on the outcomes of patients with advanced HR+/HER2− disease, improving cancer-related outcomes while maintaining a good quality of life. CDK4/6i is now the preferred first-line treatment for patients with metastatic HR+/HER2− disease.

Ribociclib is an active drug that is metabolized (into inactive metabolites) by CYP3A^12^. Therefore, the recommended ribociclib dose varies with CYP3A activity. Prior studies identified that ribociclib is metabolized by CYP3A, and concomitant use of strong CYP3A inhibitors increased ribociclib exposure by 3.2-fold^13^. The FDA recommends the use of alternative therapy or a 50% dose reduction (i.e., from 600 mg to 400 mg) if ribociclib is used with strong CYP3A inhibitors^14^. However, it is unknown if dose changes are needed based on variations in baseline CYP3A activity, which is constituted by CYP3A4 and CYP3A5.

Variations in the genes encoding these proteins, *CYP3A4* and *CYP3A5*, could mimic the effect of inhibition of these enzymes. There is currently insufficient evidence regarding the potential influence of *CYP3A4* and *CYP3A5* genotype on ribociclib exposure^15^. For *CYP3A4*, outside of rare variants (e.g., CYP3A4*22, CYP3A4*6), there is a paucity of evidence describing the broad impacts of *CYP3A4* variants on enzymatic function. In contrast, variations in *CYP3A5* genotype are well established with tacrolimus dosing and pharmacokinetic properties^16^. In brief, patients who express CYP3A5 (i.e., intermediate or normal metabolizers) are more likely to experience subtherapeutic trough levels and require higher doses of tacrolimus. Interestingly, previous evidence, including the FDA label, suggests that African American patients require higher doses of tacrolimus than White patients^16–18^. Although race and genetic ancestry are distinct, these findings align with the fact that patients of African ancestry are likely (~85%) to be expressors (normal or intermediate metabolizers) of CYP3A5^16,17,19^. This is contrary to patients of European ancestry where ~85% are CYP3A5 poor metabolizers (CYP3A5 non-expressors)^16^

CDK4/6i were approved based on large studies. However, there was limited representation of racial and ethnic minorities; for example, in the pivotal trials studying ribociclib, only 41 of 2066 (<2%) patients identified as Black^6–8^. This is primarily a concern because the lack of underrepresented patients reflects a potential continuation of disparities in care^20^. Secondly, it also suggests ribociclib dosing was tested in a population that predominantly lacks CYP3A5 expression (poor metabolizers), and other patient populations may require a different dose to achieve similar outcomes in safety and efficacy. Specifically, patients who self-identify as Black or African American were underrepresented in ribociclib studies and there may be differences in ribociclib metabolism and ribociclib exposure based on *CYP3A5* genotype, which could lead to altered clinical outcomes. LEANORA was a prospective study that assessed the pharmacogenomics (PGx) and pharmacokinetics (PK) of ribociclib in self-identified Black patients with advanced breast cancer.

## Results

### Patient population

Between May 2021 and March 2024, 84 patients were reviewed; 63 patients were ineligible (e.g., not metastatic disease, EKG abnormality) or declined to participate, three participants withdrew after providing consent (i.e., participant changed their mind [*n* = 1], inability to obtain blood samples for analyses [*n* = 2]), 3 participants enrolled in the non-Hispanic White (NHW) cohort that closed to due low accrual (Supplementary figure 1). Results, including the NHW cohort, are available in Supplementary tables 1–4. Fourteen self-identified Black participants with HR+/HER2− advanced breast cancer completed the study, and their data was included in this analysis. Demographic characteristics are described in Table 1. The median age was 61.5 years, and none of the patients identified as Hispanic. The most common sites of metastatic disease were bone, soft tissue, and lung. All patients received ribociclib and ET (letrozole or fulvestrant) per standard of care. The majority (13) of the patients received letrozole, while one received fulvestrant. Three patients received concurrent ovarian suppression.

**Table 1:** Patient characteristics

	Participants *n* (%) *N* = 14
Median age (interquartile range)	61.5 (54, 66)
Race	
African American	14 (100)
Ethnicity	
Non-Hispanic	14 (100)
Metastatic sites	
Bone	9 (64)
Soft tissue	5 (36)
Lung	3 (21)
Adrenal	1 (7)
Liver	1 (7)
Brain	0 (0)
ECOG	
0	8 (57)
1	6 (43)
Smoking History	
Active Smoker	3 (21)
Former Smoker	5 (36)
Never Smoker	6 (43)
Endocrine Therapy	
Letrozole	13 (93)
Fulvestrant	1 (7)
Ovarian Suppression Therapy	
None	11 (79)
Goserelin or leuprolide	3 (21)
Concomitant Medications	
Prohibited medication	0 (0)
Not recommended medication	3 (21)^a^
CYP3A5 Phenotype	
Normal Metabolizer (Expressor)	1 (7)
Intermediate Metabolizer (Expressor)	6 (43)
Poor Metabolizer (Non-expressor)	7 (50)

Median and interquartile range are shown for age.

^a^One participant was prescribed two medications that were not recommended (metformin continued through study; atorvastatin held D-1 through D12 after completion of PK sampling) and two participants were prescribed one medication that was not recommended (ondansetron and mirtazapine).

### CYP3A5 genotype/phenotype

A total of 50% were CYP3A5 intermediate metabolizers (IM)/ normal metabolizers (NM) (*n* = 1 NM and *n* = 6 IM) and 50% (*n* = 7) poor metabolizers (PM). The genotype was CYP3A5*1/*1 for the one NM. The IM phenotype included the CYP3A5*1/*3 (*n* = 3), CYP3A5*1/*6 (*n* = 2), and CYP3A5*1/*7 (*n* = 1) genotypes. The PM phenotype included the CYP3A5*3/*3 (*n* = 4), CYP3A5*3/*6 (*n* = 2), and CYP3A5*3/*7 (*n* = 1) genotypes.

### Human liver microsome analysis

**Fig. 1:**
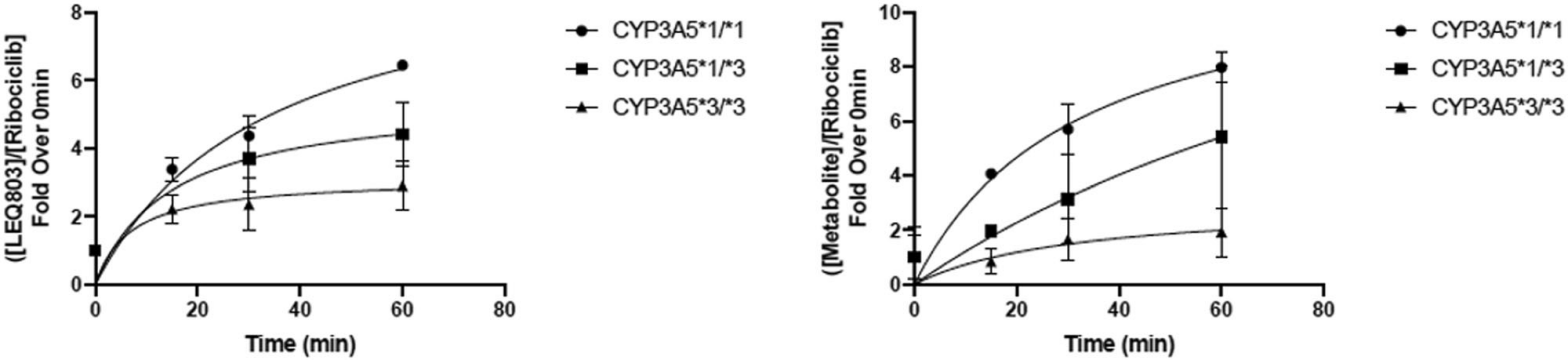
Effect of *CYP3A5* genotype on ribociclib metabolism in human liver microsomes. Formation of major ribociclib metabolites over time was correlated with *CYP3A5* genotype status. Human liver microsomes from a CYP3A5 NM (CYP3A5 *1/*1) had the greatest ribociclib metabolite formation followed by IM (CYP3A5 *1/*3) and PM (CYP3A5 *3/*3). Graphs represent three technical replicates per datapoint, error bars show standard deviation.

Ribociclib metabolism was evaluated in human liver microsomes obtained from three individuals not from this current study, each harboring a different *CYP3A5* genotype: CYP3A5*1/*1 (NM), CYP3A5*1/*3 (IM), and CYP3A5*3/*3 (no CYP3A5 function or PM). Consistent with the importance of CYP3A5 in ribociclib metabolism, the formation of major ribociclib metabolites was correlated with *CYP3A5* genotype status (Fig. 1). Microsomes from a CYP3A5 NM had the greatest ribociclib metabolite formation followed by IM and PM.

### Pharmacokinetic and pharmacogenetic findings

The primary endpoint, area under the curve (AUC_TAU_), was similar between CYP3A5 PM (39,230 h*ng/mL; IQR: 18,745 to 57,566 h*ng/mL) vs. IM/NM (43,546 h*ng/mL; IQR: 35,298 to 46,647 h*ng/mL; *p* = 0.38). PK properties by CYP3A5 phenotype are summarized in Table 2. Similarly, there were no statistical differences in maximum concentration (C_max_) or in the time to reach C_max_ suggesting that differences in *CYP3A5* genotype did not impact ribociclib exposure.

**Table 2:** Pharmacokinetics of ribociclib by CYP3A5 phenotype^a^

	CYP3A5 IM/NM *n* = 7	CYP3A5 PM *n* = 7	*P*-value
AUC_tau_ (h*ng/mL)	43,546 (35298, 46647)	39,230 (18745, 57566)	0.38
AUC_0-6 h_ (h*ng/mL)	14,907 (8254, 15245)	11,102 (6143, 13651)	0.26
C_max_ (ng/mL)	3,140 (1980, 3540)	3,020 (1300, 3470)	0.46
*T*_max_ (h)	2.0 (2.0, 4.0)	3.8 (2.0, 5.9)	0.48

*AUC* area under the curve, *C*_*max*_ maximum concentration, *IM/NM* intermediate metabolizers/normal metabolizers, *PM* poor metabolizers *T*_*max*_ time to reach maximum concentration

^a^Median and interquartile range are shown for continuous data

### Toxicity profile

Adverse events (AEs) of interest are summarized in Table 3. Overall, the toxicity profile was consistent with previous reports with ribociclib. The most common toxicities were leukopenia, nausea, vomiting, and diarrhea. The reported grade 3 toxicities included neutropenia, nausea, vomiting, transaminitis, and increased creatinine. This study was not powered to assess differences in AEs between PMs and IM/NMs. There was a similar number of all grade toxicities by CYP3A5 phenotype: 100% (7 of 7) vs. 71% (5 of 7) for IM/NM vs. PMs, respectively (*p* = 0.46). PMs also had a similar number of grade 3+ AEs (29%, 2/7) compared to IM/NMs (29%, 2/7) (*p* = 1). Table 4 shows a similar change in laboratory values (i.e., transaminases, absolute neutrophil count [ANC]) and QTc by CYP3A5 phenotype between baseline and mid-cycle and end of cycle 1. Supplementary table 3 provides data about the toxicity profile in the entire study, and Supplementary table 4 provides additional AE data related to QTc prolongation, including one case from cohort 2 where a participant experienced grade 1 QTc prolongation and was unknowingly prescribed a prohibited medication that could prolong the QTc (venlafaxine). Univariate analyses of clinical factors did not identify significant associations with ribociclib AUC (Table 5). These findings should be considered with caution due to the small sample size and because the study was not powered to assess these differences.

**Table 3:** Adverse events of interest by CYP3A5 phenotype

	CYP3A5 IM/NM *n* = 7		CYP3A5 PM *n* = 7	
	Toxicity (any grade)	Toxicity (grade 3+)	Toxicity (any grade)	Toxicity (grade 3+)
**Any AE**	7	2	5	2
**Hematologic**				
Neutropenia	0	0	3	1
Thrombocytopenia	1	0	0	0
Leukopenia	3	1	2	1
Anemia	2	1	0	0
**Gastrointestinal**				
Diarrhea	1	0	2	0
Nausea	3	1	2	1
Vomiting	2	1	2	1
Transaminitis	1	0	1	1
Constipation	1	0	1	0
Abdominal pain	0	0	1	0
Melena	1	0	0	0
Mucositis	1	0	0	0
**Cardiac**				
QTc Prolongation	3	0	1	0
Palpitations	1	0	0	0
Sinus tachycardia	1	0	0	0
**Metabolism and nutrition disorders**				
Arthralgia	1	0	1	0
Back pain	0	0	1	0
Bone pain	1	0	0	0

The list of specific types of AEs has been abbreviated. A complete list of recorded adverse events are available in Supplementary table 3.

*AE* adverse events, *IM/NM* intermediate metabolizers/normal metabolizers, *PM* poor metabolizers

**Table 4:** Association between CYP3A5 phenotype and laboratory or electrocardiogram values

Lab Test	Difference at mid-cycle of C1		SD	*p* value	Difference at end of C1		SD	*p* value
	CYP3A5 IM/PM	CYP3A5 NM			CYP3A5 IM/PM	CYP3A5 NM		
ALT (U/L)	−1 (−8, 2)	−4 (−9, 4)	−0.21	0.78	−7 (−10, 3)	−5 (−9, 6)	−0.36	0.56
AST (U/L)	0 (−5, 2)	−2 (−6, 3)	0.54	0.71	−2 (−6, 5)	−2 (−6, 13)	0.51	0.88
QTc (ms)	9 (−5, 20)	18 (−2, 46)	0.07	0.54	3 (−1, 28)	16 (−21, 27)	−0.45	0.80
ANC (1000/mm^3^)	−2 (−2, 0)	−3 (−4, −1)	−1.1	0.09	−2 (−2, −1)	−3 (−6, −2)	−1.1	0.10

*ALT* alanine aminotransferase, *ANC* absolute neutrophil count, *AST* aspartate aminotransferase, *C1* cycle 1, *IM/NM* intermediate metabolizers/normal metabolizers, *PM* poor metabolizers, SD: standard deviation

Median and interquartile range are shown for continuous data.

SD represents the standardized difference in CYP3A5 poor metabolizers minus the CYP3A5 IM/PM for the change in each endpoint (post minus baseline).

**Table 5:** Univariate analysis of clinical factors on ribociclib AUC

	Level	*n*	Statistics	*p*-value^a^
			Correlation^a^	
Age (years)	–	14	0.43	0.1258
Weight (kg)	–	14	−0.28	0.3253
Body surface area	–	14	−0.34	0.2360
Creatinine clearance (mL/min)	–	14	−0.25	0.3825
Days of ribociclib therapy	–	14	−0.03	0.9153
			median	
ECOG performance status^b^	0	8	39200	0.5728^c^
	1	6	43430	
Smoking	Never	6	41155	0.4867^d^
	Former	5	32798	
	Current	3	46647	
	Never/former	11	39230	0.0879^c^
	Current	3	46647	
Concomitant unrecommended medication^e^	0	11	43081	0.6612^d^
	1	2	35679	
	2	1	57566	

^a^Spearman’s correlation and the corresponding p-value

^b^0: fully functional, 1: limited functional

^c^An exact Wilcoxon rank sum test-based p-value

^d^An exact Jonckheere-Terpstra trend test-based p-value

^e^The number of unrecommended medications taken by each participant. The two participants taking one unrecommended medication were prescribed a medication that may prolong QT interval (mirtazapine, ondansetron). The participant with two unrecommended medications was prescribed metformin (ribociclib may increase metformin levels) throughout the study and atorvastatin (CYP3A substrate) was paused the day before ribociclib initiation until D13 after completion of sample collection for PK analysis.

*AUC* area under the curve

### Exploratory candidate gene analysis

**Fig. 2:**
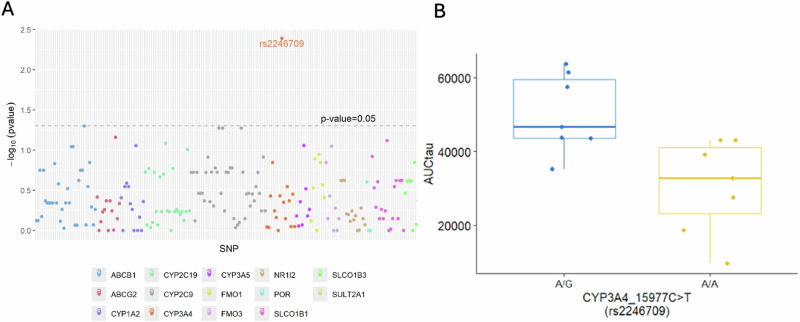
*P*-values by different SNPs of interest and AUCtau by rs2246709. **2A** Genetic data were available for all 14 exploratory candidate gene analysis participants. Variants are plotted by *p*-value with a dotted line representing a *p*-value of 0.05. This exploratory analysis of 14 participants identified one variant with a *p* < 0.05 for ribociclib AUC_TAU_: CYP3A4 rs2246709 (*p* = 0.0041). **2B**. This exploratory analysis identified an association between ribociclib area under the curve (AUC) in participants with a *CYP3A4* variant (rs2246709), showing a higher AUC in those with A/G relative to those with A/A (*p* = 0.0041).

Genetic data were available for all 14 participants in the exploratory candidate gene analysis. Out of 495 variants assessed, 285 variants were identical among all participants. No further evaluation was conducted among those 285 variants. The remaining 210 variants yielded two different genotypes in 106 variants and three different genotypes in 104 variants. This exploratory analysis of 14 participants identified one variant with a *p* < 0.05 for ribociclib AUC_TAU_: *CYP3A4* rs2246709 (*p* = 0.0041), with A/G being associated with elevated AUC_TAU_ compared to A/A (Fig. 2A, B).

## Discussion

In the LEANORA trial, we found no association between *CYP3A5* genotype and ribociclib exposure or adverse events among self-identified Black participants. Our findings do not suggest that *CYP3A5* screening is warranted to determine the safety of ribociclib. However, correlative studies of larger trials using ribociclib may provide more insight into interindividual exposure and response to this agent. This was seen in the microsome data in which metabolites from a CYP3A5 NM had the greatest ribociclib metabolite formation (inactive) followed by IM and PM, respectively.

These findings are aligned with prior population pharmacokinetics (popPK) and pharmacokinetic/pharmacodynamic (PK/PD) models^21^. We identified weak associations between age and increased ribociclib AUC_TAU_ and weight and decreased AUC_TAU_. This small inverse correlation between body weight and ribociclib exposure is aligned with data included in the FDA review of ribociclib but is not expected to be clinically relevant^22^. Similar to ribociclib, palbociclib is predominantly metabolized by CYP3A^15^. A case report identified that CYP3A5 expression (CYP3A5*1/*3, IM) was associated with lower palbociclib plasma concentration^23^. James et al. conducted several studies that identified ~63% of ribociclib metabolism is produced by CYP3A^24^. There is significant overlap in the substrates of CYP3A4 and CYP3A5, and it is possible that both impact drug metabolism^13,24^. However, our findings suggest *CYP3A5* genotype has a minimal role. Recombinant human enzyme studies suggest CYP3A4 is the primary enzyme responsible for ribociclib metabolism^24^.

It is possible that genetic variations in *CYP3A4* may be associated with ribociclib pharmacokinetics, but variations in *CYP3A4* are less common. One pre-identified *CYP3A4* variant of interest, CYP3A4*22, was present in one participant in this trial, which limited further investigation of that specific variant. Additionally, the exploratory candidate gene analysis identified one variant of *CYP3A4*, rs2246709, potentially associated with ribociclib AUC_TAU_, which happened to be located in *CYP3A4*. However, this is an intronic variant with no obvious impact on enzyme expression or function. A query in the National Institutes of Health (NIH) LDLink did not identify linkage disequilibrium with other variants (highest R^2^ was 0.36)^25^. Future steps could include exploring the impact of rare variants on ribociclib exposure in larger samples or may also explore genetic associations (e.g., *KCNH2*, *SCN5A*, *SNTA1*) with QTc prolongation secondary to ribociclib therapy^26^.

This study was focused on identifying a potential association between *CYP3A5* genotype and ribociclib PKs. Therefore, concomitant medications were strictly regulated. In the one case where a patient was unknowingly prescribed a prohibited medication (venlafaxine), administration of this agent was associated with an adverse event (Grade 1 QTc prolongation). The retrospective AB-ITALY study, included 173 patients treated with abemaciclib and endocrine therapy and revealed that software-predicted CDK4/6 drug interactions were an independent predictor of worse PFS^27^. The findings of this study are hypothesis-generating and serve as an important reminder for clinicians and pharmacists to monitor patient’s concurrent medications when prescribing a CDK4/6 inhibitor.

In terms of ribociclib dosing, the current recommended dose for patients with mBC is 600 mg daily for 21 days, followed by 7-day off treatment. There are prespecified dose reductions based on tolerance. An analysis of the MONALEESA 2, 3, and 7 studies revealed that 42% of patients required a dose reduction, which did not affect the efficacy of the drug^28^. The AMALEE (NCT03822468) study compared the standard 600 mg dose of ribociclib to 400 mg with the same schedule; after a median follow-up of 14 months, the study did not meet its primary endpoint of non-inferiority for the low dose based on objective response rate^29^. However, the study showed a favorable toxicity profile of the lower dose, particularly for neutropenia and QTc prolongation. The approval of cancer-directed therapies has traditionally been based on the maximum tolerated dose, and in recent years, there has been a shift to consider optimal biological doses. The latter is not based on toxicities but rather on other cancer-specific endpoints such as AMALEE^30^. We encourage researchers to rethink study endpoints to improve patient outcomes while limiting toxicity when possible.

Although no difference was found in ribociclib pharmacokinetics by *CYP3A5* genotype, this trial has important implications for clinical practice. It is reassuring for patients and clinicians that the standard of care is not affected by the *CYP3A5* genotype. Additionally, this trial represents the largest known cohort of ribociclib PK data in patients who self-identify as Black.

It is critical to understand the differences between race and ancestry when conducting and interpreting research. Race and ethnicity are social constructs that can be self-ascribed and based on shared physical or social qualities^31^. They do not necessarily reflect genetic ancestry; therefore, using race or ethnicity as proxies for genetic ancestry can be inaccurate^19^. Genetic ancestry refers to people in the past to whom an individual is biologically connected^32,33^. Genetic ancestry and genealogy can influence frequencies of genetic variants (e.g., carrier of HLA-B*15:02 or CYP3A5*1). Recommending genetic testing based on race or ethnicity remains controversial^33–35^. It is likely that race, ethnicity, and genetic ancestry all contribute to patient outcomes. This trial studied an underrepresented population based on race as well as genetic variants more prevalent among those of African ancestry.

Strengths of this trial include that it was a PK and PGx study conducted in participants with cancer and to our knowledge, this is the largest cohort with ribociclib pharmacokinetic data in Black participants. Lastly, the robust sample collection (prior to the ribociclib dose, and 0.5 h, 1 h, 2 h, 4 h, and 6 h) allows for calculation of AUC over a 24-hour period given the reported T_max_ is one to four hours per the FDA label^14^.

Limitations of this study include limited sample size for secondary analyses (e.g., adverse events) and for the investigation of rare variants on ribociclib pharmacokinetics. Secondly, it only followed participants through the first cycle, data on long term toxicities and the impact of dose reductions in PKs are not available.

In conclusion, we found no association between *CYP3A5* genotype and ribociclib exposure or adverse events through cycle 1. Ensuring diverse patient representation in clinical trials is critical results that are applicable to the population, so we can continue to improve outcomes of all patients.

## Methods

### Study design

LEANORA (NCT04657679) was a prospective, observational, multicenter cohort study that assessed the pharmacokinetics and pharmacogenomics of ribociclib. The trial was opened in four sites in the United States (Georgetown Lombardi Comprehensive Cancer Center, Washington DC; MedStar Washington Hospital Center, Washington DC; Tufts Medical Center, Boston, MA; MedStar Franklin Square, Baltimore MD). All participants were followed for the first cycle and prescribed ribociclib 600 mg daily (3 weeks on, 1 week off) plus letrozole 2.5 mg daily or fulvestrant 500 mg on days 1 and 15. Ovarian suppression was required for premenopausal patients, with either goserelin (3.6 mg every 28 days or 10.8 mg every 12 weeks) or leuprolide depot (3.75 mg every 28 days or 11.25 mg every 12 weeks). Dose modifications were provided for ribociclib based on adverse events; however, none of the participants had dose reductions prior to the PGx and PK studies. The institutional review boards (IRBs) approved the study at Georgetown University -and affiliated sites- (study ID: STUDY00003100) and Tufts Medical Center (study ID: STUDY00002025). Written informed consent was obtained from all participants. All procedures were in accordance with the ethical standards of the Declaration of Helsinki.

### Patient population

Eligible patients included women with previously untreated HR+/HER2− metastatic or locally advanced breast cancer who self-identified as NHW or African American/Black. Documentation of estrogen receptor (ER) positive and/or progesterone receptor (PR) positive tumor (≥1% positive stained cells) based on the most recent tumor biopsy utilizing an assay consistent with local standards. HER2 negative was defined by the American Society of Clinical Oncology/College of American Pathologists (ASCO/CAP) guidelines^36^. Patients were excluded if they were receiving other ET (given for concern for potential drug interaction or increased risk for toxicity) or if they were receiving concurrent medications that could impact CYP3A, other proteins related to ribociclib pharmacokinetics, or risk of adverse events (Supplementary tables 5-6).

Participants were considered postmenopausal if (i) they have had prior bilateral oophorectomy; (ii) age ≥ 60 years; (iii) age <60 years and have had amenorrhea for 12 or more months (in the absence of chemotherapy, tamoxifen, toremifene, or ovarian suppression) and follicle-stimulating hormone (FSH) and estradiol in the postmenopausal range per local normal ranges.

The study initially enrolled participants into two independently powered cohorts based on self-identified race: cohort 1) African American or Black participants and cohort 2) NHW participants. However, the planned cohort 2 only enrolled three patients, and due to difficulty enrolling patients, this cohort was closed. In this manuscript, we present the data about cohort 1. However, the results of the patients enrolled in cohort 2 are described in the Supplementary material.

### Human liver microsome analysis

Human Liver Microsomes (20 pmol/mL CYP/reaction; 37°C, Seksui Xenotech, Kansas City, KS) were added to preincubated reaction buffer containing 0.5 M potassium phosphate buffer (pH 7.4), NADPH solutions A and B according to the manufacturer’s instructions (Corning, Corning NY), and ribociclib (Selleckchem, Houston, TX) or tacrolimus (MedChemExpress, Monmouth Junction, NJ) dissolved in acetonitrile at a final concentration of 25 µm (0.1% acetonitrile). Aliquots (100 μL) were removed from the reaction immediately after the addition of HLMs (0 min) and 60 min later. Reactions were terminated by the addition of 100 μL of ice-cold acetonitrile followed by 1 min of vortexing. Tubes were then centrifuged at maximum speed for 10 min at 4°C and harvested supernatant was stored (−20°C) until pharmacokinetic analysis. Bioanalytical measurements of ribociclib and metabolite concentrations were made using an LC-MS/MS assay described in the next section.

### Pharmacokinetic and pharmacogenetic testing

**Fig. 3:**
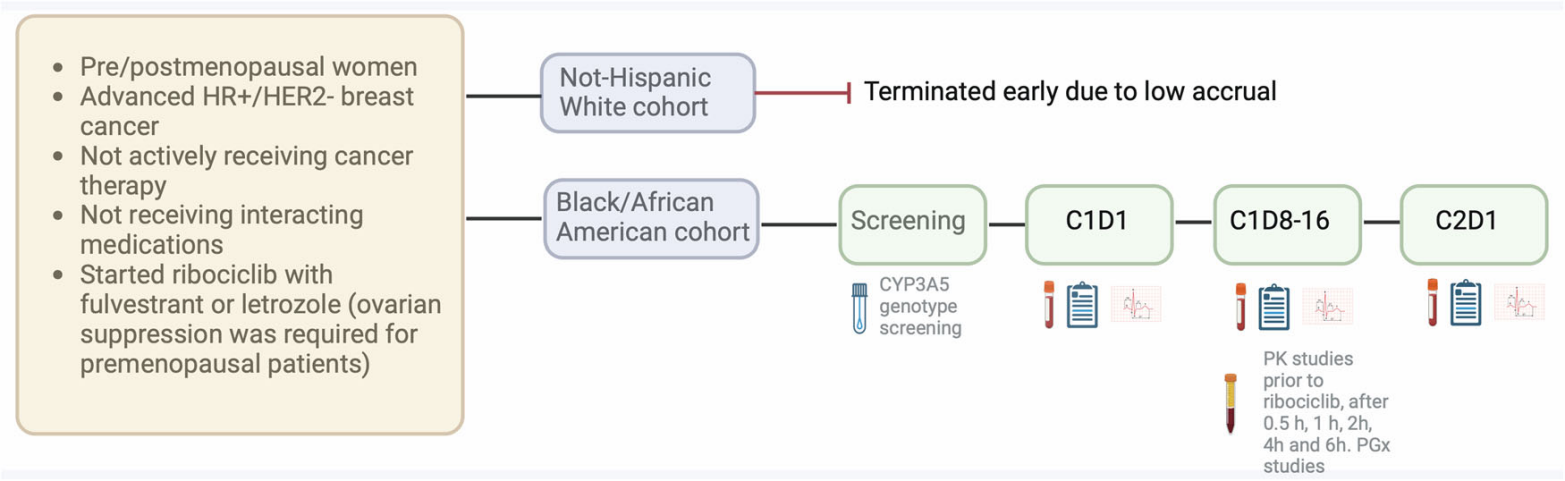
Study design. During screening, a swab *CYP3A5* genotype was obtained to ensure adequate power. On C1D1, C1D8-16, and C2D1, blood counts and chemistries were obtained, as well as an electrocardiogram per standard of care. The list of concurrent medications was also obtained, and patients received a drug diary and patient-reported outcomes (CTCAE-PRO) questionnaires. Created with BioRender.com.

Serial blood samples were collected for PK and PGx analyses (Fig. 3). The FDA label reports that ribociclib has an elimination half-life of 32 h and a maximum plasma concentration (*C*_max_) of 1 to 4 h^14^. For sampling to occur at steady-state concentrations, the collection day was scheduled between days 8 and 16 of therapy during cycle 1. Blood samples were collected immediately prior to the ribociclib dose, and 0.5 h ± 5 min, 1 h ± 5 min, 2 h ± 15 min, 4 h ± 15 min, and 6 h ± 15 min after the daily dose of ribociclib, processed to plasma and stored at −80 °C until bioanalysis.

For in vitro metabolism experiments and clinical PK plasma samples, ribociclib total concentrations (i.e. protein bound+unbound) were measured using a validated and robust LC-MS/MS assay using palbociclib as an internal standard. Briefly, samples were mixed with acetonitrile containing palbociclib as an internal standard and run alongside calibration and quality control (QC) standards, ranging from 0.25 to 50 μM. The analyte (ribociclib) and internal standard were chromatographically separated using a Phenomenex Polar Omega C18 column (5 μm, 2.1 × 100 mm) on a Shimadzu Prominence HPLC system (Shimadzu, Columbia, MD). A gradient mobile phase consisting of 0.1% formic acid (aq) and 0.1% formic acid in acetonitrile was flowed at a rate of 0.4 mL/min; the injection volume was 5 μL. Following chromatographic separation and elution, the compounds were detected using tandem mass spectrometric detection in the positive ion mode. MRM transitions were *m/z* 435.3 → 322.2 and *m/z* 448.4 → 380.2 for ribociclib and palbociclib, respectively. Additionally, ribociclib metabolites were also detection and quantitated based off the parent ribociclib calibration: *m/z* 421.3 → 322.2 (Demethyl); m/z 407.3 → 322.2 (Di-demethyl); *m/z* 451.3 → 338.3 (Hydroxyl); *m/z* 449.3 → 336.3 (Oxidation).

A non-compartmental approach to clinical plasma PK analysis was employed using Phoenix WinNonlin v8.3 (Certara Corp, Cary, NC) that was validated per FDA 21CFR Part 11 regulations. The maximum observed plasma concentration (*C*_max_) and the time of *C*_max_ (*T*_max_) were recorded as observed values. The area under the concentration-time curve over the first 6 h post-dose and over the dosing interval during steady-state (AUC_0-6 h_ and AUC_tau_, respectively) was calculated using the linear-up/log-down trapezoidal method.

Ten milliliters of frozen whole blood was used to isolate DNA via the Wizard® Genomic DNA Purification Kit (Promega) according to manufacturer instructions. PGx data were obtained from the PharmacoScan™ (ThermoFisher) microarray per manufacturer instructions, which tests 4,627 pharmacogenetic markers in 1,191 genes. This test included the three variants known to correspond with no function (i.e., CYP3A5*3, *6, and *7). In accordance with Clinical Pharmacogenetics Consortium (CPIC) guidelines, Phenotypes assigned: poor metabolizers (PM, 2 variant alleles), intermediate metabolism (IM; 1 variant allele), NM (0 variant alleles)^16^.

### Endpoints

The primary endpoint was ribociclib AUC_tau_ (visit between days 8-16 of cycle 1) CYP3A5 PM and CYP3A5 IM/NM. Secondary endpoints included additional ribociclib pharmacokinetic properties and adverse events (AEs). Pharmacokinetic properties were maximum concentration (*C*_max_), AUC_0-6 h_, the time to reach *C*_max_ (*T*_max_), clearance, volume of distribution(v_d_), and elimination half-life. Secondary safety endpoints included change in QTc interval and occurrence of neutropenia or aspartate aminotransferase (AST) or alanine aminotransferase (ALT) elevations per Common Terminology Criteria for Adverse Events (CTCAE) version 5.0. An appearance or worsening of an undesirable sign, symptom, or medical condition after initiation of ribociclib per the CTCAE was considered an adverse event regardless of attribution to ribociclib.

### Sample size calculation

Sample size accounted for *CYP3A5* allelic frequencies for *CYP3A5* from CPIC and PharmGKB, which were translated to phenotypes via the Hardy-Weinberg equation^16^. Based on known frequencies for African Americans (normal metabolism: 35%; intermediate metabolism: 48%; poor metabolism: 16%), a sample size of 18 would provide 80% power at an α = 0.05 to detect a 2-fold change in AUC (SD is 0.56) between CYP3A5 PM vs. CYP3A5 expressors (i.e., CYP3A5 IM/NM). After the enrollment of 14 participants who self-identified as African American or Black, the distribution of CYP3A5 results was a 1:1 split for CYP3A5 IM/NM vs. PM. A second sample size calculation identified this cohort would provide 86.5% power to detect a 2 vs 1 difference in AUC pending the SD is 0.56. Alpha set at 0.05. The cohort that enrolled self-identified non-Hispanic White participants was terminated prematurely due to low accrual (*n* = 3). A buccal swab for *CYP3A5* genotyping (Kailos Genetics) was obtained during the screening period for each patient to ensure adequate sample sizes of each phenotype per cohort.

### Statistical analysis

The area under the curve (AUC_tau_) between CYP3A5 PMs vs. IM/NMs was compared with the exact Wilcoxon rank-sum test due to small sample sizes, with alpha set to 0.05. Secondly, a multiple regression analysis was intended to identify variables associated with ribociclib AUC. Covariates to be assessed include CYP3A5 metabolism status, CYP3A4 metabolism status, renal function, liver function, age, race, weight, sex, use of “medication not recommended”, AI, number of ribociclib doses taken. However, the multiple linear regression was not conducted due to the inability to provide valid inference with the small sample size. Fisher’s exact test assessed the AEs and grade 3+ AEs to day 28. Descriptive statistics were used to characterize the data profile, frequency, and percentages for categorical variables and mean (SD) or median [IQR] for continuous variables based on the data normality. Laboratory and electrocardiographic assessments were done per the current standard of care at baseline, close to C1D15, and at the end of the first cycle^14^. The QT interval was corrected using the Bazett method. Analysis was performed using SAS™ software (v 9.1; Cary, NC, USA) and R Statistical Software (v4.3.1; R Core Team 2023).

### Exploratory candidate gene analysis

We conducted an exploratory candidate gene analysis among measured variants in genes that were previously linked to ribociclib pharmacokinetics. The objective of this analysis was to assess if there was a strong genetic effect on ribociclib AUC_TAU_. A literature review in May 2024 (search terms “ribociclib AND pharmacokinetics” in PubMed) identified 80 articles. Articles were screened for content related to genes or proteins related to ribociclib metabolism. Seventeen articles met inclusion and led to the identification of 15 genes of interest: *ABCB1, ABCG2, CYP1A2, CYP2C19, CYP2C9, CYP3A4, CYP3A5, FMO1, FMO3, NR1/2, PPARα, POR, SLCO1B1, SLCO1B3, SULT2A1*. Genetic data were available for 14 of 15 genes, with it unavailable for *PPARα*. The list of variants interrogated is available in Supplementary Table 7. The association of each of the variants with ribociclib AUC_TAU_ was evaluated by an exact Wilcoxon rank sum test or a Kruskal-Wallis test if there are two or three types of genotype within each variant, respectively. All the statistical results should be interpreted as exploratory and descriptive with two-tailed p-values unadjusted for multiple comparisons.

## Supplementary information

Supplementary data

## Data Availability

Deidentified data used for this analysis are publicly available at dbGAP (https://www.ncbi.nlm.nih.gov/gap/) with data accession code phs003770.v1.p1.
