# Supplementary material for: Pharmacokinetics and pharmacogenomics of ribociclib in black patients with metastatic breast cancer the LEANORA study
Source: NPJ Breast Cancer. 2024 Sep 30;10:84. doi: 10.1038/s41523-024-00692-w (PMC11442496; doi:10.1038/s41523-024-00692-w)
Supplement: Supplementary file 1 — Supplementary data [file 41523_2024_692_MOESM1_ESM.docx]

**Supplementary material**

**Supplementary figure 1:** Consort diagram of participant enrollment and follow-up

**
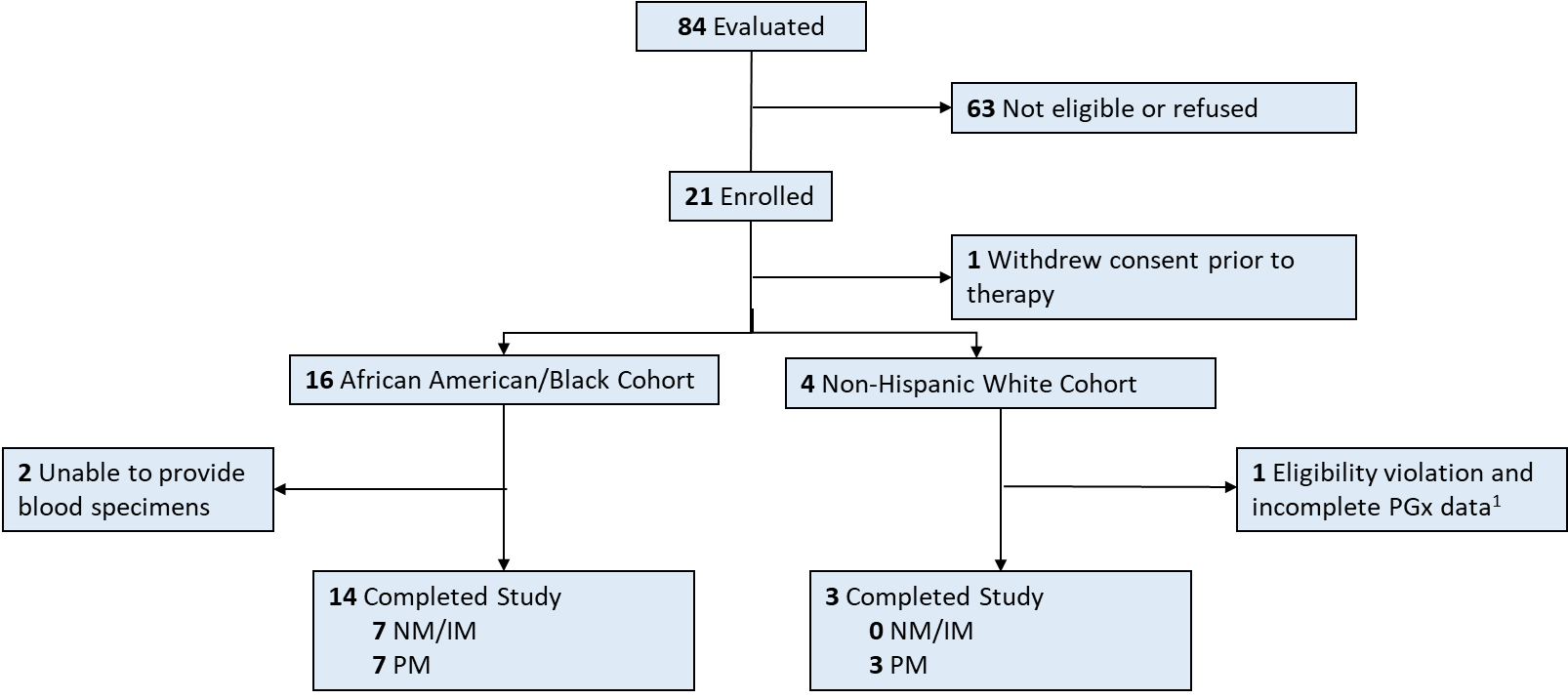
**

^1^During analysis it was identified that this participant was an eligibility violation due to concomitant therapy of a prohibited medication (i.e., venlafaxine), which placed the participant at increased risk of QTc prolongation. This participant was in the White Non-Hispanic Cohort.
Abbreviations: IM/NM: intermediate metabolizers/normal metabolizers; PGx: pharmacogenomics; PM: poor metabolizers

**Supplementary Table 1**: Participant characteristics of all who initiated ribociclib

| **Characteristic** | **Participants n (%)**  **N = 20** |
| --- | --- |
| Median age (range) | 62.5 (54, 68) |
| Race |  |
| African American | 14 (70) |
| White | 6 (30) |
| Ethnicity |  |
| Non-Hispanic | 20 (100) |
| Metastatic sites |  |
| Bone | 11 (55) |
| Soft tissue | 5 (25) |
| Lung | 6 (30) |
| Liver | 2 (10) |
| Adrenal | 1 (5) |
| Brain | 0 (0) |
| ECOG |  |
| 0 | 12 (60) |
| 1 | 8 (40) |
| Smoking History |  |
| Active Smoker | 3 (15) |
| Former Smoker | 7 (35) |
| Never Smoker | 10 (50) |
| Endocrine Therapy |  |
| Letrozole | 19 (95) |
| Fulvestrant | 1 (5) |
| Ovarian Suppression Therapy |  |
| None | 15 (80) |
| Goserelin or leuprolide | 5 (20) |
| Concomitant Medications |  |
| Prohibited medication | 1 (5)^1^ |
| Not recommended medication | 3 (15)^2^ |
| CYP3A5 Phenotype |  |
| Normal Metabolizer (Expressor) | 1 (5) |
| Intermediate Metabolizer (Expressor) | 7 (35) |
| Poor Metabolizer (Non-expressor) | 12 (60) |

Median and interquartile range are shown for age.
^1^During analysis it was identified that this participant was an eligibility violation due to concomitant therapy of a prohibited medication (i.e., venlafaxine), which placed the participant at increased risk of QTc prolongation. This participant was in the White Non-Hispanic Cohort.

^2^One participant was prescribed two medications that were not recommended (metformin continued through study; atorvastatin held D-1 through D12 after completion of PK sampling) and two participants were prescribed one medication that was not recommended (ondansetron and mirtazapine).

**Supplementary Table 2:** Ribociclib pharmacokinetics by CYP3A5 phenotype among all participants who completed the mid-cycle visit of cycle 1

| **Pharmacokinetic Properties** | **African American/Black Cohort** | | **Non-Hispanic White Cohort** | |
| --- | --- | --- | --- | --- |
|  | **CYP3A5 IM/NM n=7** | **CYP3A5 PM n=7** | **CYP3A5 IM/NM n=0** | **CYP3A5 PM n=3** |
| AUC_tau_ (hr*ng/mL) | 43546 (35298, 46647) | 39230 (18745, 57566) | - | 33230 (17142, 39492) |
| AUC_0-6hr_ (hr*ng/mL) | 14907 (8254, 15245) | 11102 (6143, 13651) | - | 11172 (6623, 11893) |
| C_max_ (ng/mL) | 3140 (1980, 3540) | 3020 (1300, 3470) | - | 2300 (2220, 2640) |
| T_max_ (hr) | 2.0 (2.0, 4.0) | 3.8 (2.0, 5.9) | - | 0.97 (0.58, 4.1) |

*Median and interquartile range are shown for continuous data.

**Abbreviations:** AUC: area under the curve, C_max_: maximum concentration, IM/NM: intermediate metabolizers/normal metabolizers; PM: poor metabolizers; T_max_: time to reach maximum concentration

**Supplementary Table 3:** Adverse events in cycle 1 by CYP3A5 phenotype among all participants with data after ribociclib initiation.

|  | **CYP3A5 IM/NM n=7** | | **CYP3A5 PM n=12** | |
| --- | --- | --- | --- | --- |
|  | **Toxicity (any grade)** | **Toxicity (grade 3+)** | **Toxicity (any grade)** | **Toxicity (grade 3+)** |
| **Any AE** | 7 | 2 | 10 | 3 |
| **Hematologic** |  |  |  |  |
| Neutropenia | 0 | 0 | 4 | 2 |
| Thrombocytopenia | 1 | 0 | 0 | 0 |
| Leukopenia | 3 | 1 | 2 | 1 |
| Anemia | 2 | 1 | 0 | 0 |
| **Gastrointestinal** |  |  |  |  |
| Diarrhea | 1 | 0 | 4 | 0 |
| Nausea | 3 | 1 | 5 | 1 |
| Vomiting | 2 | 1 | 3 | 1 |
| Transaminitis | 1 | 0 | 1 | 1 |
| Constipation | 1 | 0 | 1 | 0 |
| Abdominal pain | 0 | 0 | 1 | 0 |
| Melena | 1 | 0 | 0 | 0 |
| Mucositis | 1 | 0 | 0 | 0 |
| Oral Pain | 0 | 0 | 1 | 0 |
| **Cardiac** |  |  |  |  |
| QTc Prolongation | 3 | 0 | 4 | 0 |
| Palpitations | 1 | 0 | 0 | 0 |
| Sinus tachycardia | 1 | 0 | 0 | 0 |
| **Renal and Urinary Disorders** |  |  |  |  |
| Creatinine, increased | 2 | 0 | 2 | 0 |
| Acute kidney injury | 0 | 0 | 1 | 1 |
| **Metabolism and Nutrition Disorders** |  |  |  |  |
| Anorexia | 0 | 0 | 1 | 0 |
| Hyperglycemia | 1 | 0 | 0 | 0 |
| Hypoalbuminemia | 0 | 0 | 1 | 0 |
| Hypocalcemia | 1 | 0 | 0 | 0 |
| **Metabolism and Nutrition Disorders** |  |  |  |  |
| Arthralgia | 1 | 0 | 2 | 0 |
| Back pain | 0 | 0 | 1 | 0 |
| Bone pain | 1 | 0 | 0 | 0 |
| **Infections and infestations** |  |  |  |  |
| Gum infection | 0 | 0 | 1 | 0 |
| Infection, COVID-19 | 0 | 0 | 1 | 0 |
| Pharyngitis | 0 | 0 | 1 | 0 |
| **Respiratory, Thoracic, and Mediastinal Disorders** |  |  |  |  |
| Epistaxis | 1 | 0 | 0 | 0 |
| Pleural effusion | 1 | 0 | 0 | 0 |
| **Skin and Subcutaneous Tissue Disorders** |  |  |  |  |
| Alopecia | 0 | 0 | 2 | 0 |
| Hyperhidrosis | 2 | 0 | 0 | 0 |
| Dry skin | 1 | 0 | 0 | 0 |
| **Vascular Disorders** |  |  |  |  |
| Hot flashes | 0 | 0 | 1 | 0 |
| Hypotension | 1 | 0 | 0 | 0 |
| **Other** |  |  |  |  |
| Headache | 1 | 0 | 2 | 0 |
| Edema limbs | 1 | 0 | 1 | 0 |
| Fatigue | 1 | 0 | 1 | 0 |
| Alkaline phosphatase, increased | 0 | 0 | 1 | 0 |

This table includes data from 19 participants enrolled in LEANORA who had data collected after treatment initiation. One additional participant is excluded from this table because they withdrew after treatment initiation, but it was prior to additional data collection at the mid cycle visit. For those with incomplete PGx data (n=2), the CYP3A5 screening phenotype per a targeted next generation sequencing test was used for this table.

**Abbreviations**: AE: adverse events; IM/NM: intermediate metabolizers/normal metabolizers; PM: poor metabolizers

**Supplementary Table 4:** Changes in QTc over cycle 1 among all participants with data after ribociclib initiation.

| **Participant**^1^ | **Baseline QTc** | **Mid-cycle QTc** | **End of study QTc** | **AE Grade** | **Attribution** |
| --- | --- | --- | --- | --- | --- |
| 1 | 415 | 463 | 449 | AE – Grade 1 | Possible |
| 2 | 416 | 432 | 420 | None | N/A |
| 3 | 394 | 409 | 399 | None | N/A |
| 4^2^ | 474 | 467 | 457 | None | N/A |
| 5 | 428 | 438 | 422 | None | N/A |
| 6 | 432 | 458 | 452 | AE – Grade 1 | Probable |
| 7 | 446 | 465 | 420 | AE – Grade 1 | Possible |
| 8 | 436 | 473 | 447 | AE – Grade 1 | Possible |
| 9^2^ | 457 | 449 | 438 | None | N/A |
| 10^3^ | 453 | 453 | 464 | None | N/A |
| 11 | 408 | 408 | 437 | None | N/A |
| 12 | 399 | 431 | 352 | None | N/A |
| 13 | 443 | 429 | 439 | None | N/A |
| 14^2^ | 455 | 444 | 444 | None | N/A |
| 16 | 435 | 440 | 484 | AE – Grade 1 | Possible |
| 17 | 438 | 461 | 469 | AE – Grade 1 | Possible |
| 18 | 397 | 433 | 420 | None | N/A |
| 19 | 397 | 420 | 437 | None | N/A |
| 20^4^ | 424 | 503 | 418 | AE – Grade 1 | Probable |

^1^Data are presented for 19 patients. Patient ID 15 withdrew prior to midcycle data collection.

^2^Elevated at baseline but decreased after drug administration.

^3^Elevated (> 450 ms) at baseline but the change from baseline was considered within natural variability of the instrument per cardiologist blind to the genetic data.

^4^During analysis it was identified that this participant was an eligibility violation due to concomitant therapy of a prohibited medication (i.e., venlafaxine), which placed the participant at increased risk of QTc prolongation. This participant was in the White Non-Hispanic Cohort.

**Supplementary Table 5:** Prohibited Medications

| Category | Drug name |
| --- | --- |
| Strong inhibitors of CYP3A4/5 | Boceprevir, clarithromycin, cobicistat, conivaptan, danoprevir/ritonavir, eltegravir/ritonavir, grapefruit juice, indinavir/ritonavir, itraconazole, ketoconazole, lopinavir/ritonavir, mibefradil, nefazodone, nelfinavir, posaconazole, ritonavir, saquinavir, saquinavir/ritonavir, telaprevir, telithromycin, tipranavir/ritonavir, troleandomycin, voriconazole |
| Strong inducers of CYP3A4/5 | Avasimibe, carbamazepine, mitotane, phenobarbital, phenytoin, rifabutin, rifampin (rifampicin) , St. John's wort (hypericum perforatum) |
| Substrates of CYP3A4/5 | Alfentanil, apixaban (doses >2.5 mg only), aprepitant, astemizole, cisapride, cyclosporine, diergotamine, dihydroergotamine, ergotamine, fentanyl, lovastatin, nicardipine, nisoldipine, pimozide, quinidine, rivaroxaban, simvastatin, sirolimus, tacrolimus, terfenadine, thioridazine |
| Medications with a known risk or possible for QT prolongation and/or Torsades de Pointe (TdP) | Amiodarone, anagrelide, arsenic trioxide, astemizole, azithromycin, bepridil, chloroquine, chlorpromazine, cilostazol, ciprofloxacin, cisapride, citalopram, clarithromycin, disopyramide, dofetilide, domperidone, donepezil, dronedarone, droperidol, erythromycin, escitalopram, flecainide, fluconazole, halofantrine, haloperidol, ibutilide, levofloxacin, levomethadyl, mesoridazine, methadone, moxifloxacin, ondansetron (i.v. only), pentamidine, pimozide, probucol, procainamide, propofol, quinidine, sevoflurane, sotalol, sparfloxacin, sulpiride, terfenadine, thioridazine, vandetanib, venlafaxine |
| Herbal medications/preparations or dietary supplements that are strong inhibitors or inducers of CYP3A4/5 or those with a known risk of QT prolongation | Herbal preparations/medications are prohibited throughout the study. These herbal medications include, but are not limited to: St. John’s wort, Kava, ephedra (ma huang), gingko biloba, dehydroepiandrosterone (DHEA), yohimbe, saw palmetto, black cohosh, and ginseng. Patients should stop using these medications 7 days prior to the first dose of study drug. |

**Supplementary Table 6:** Medications not Recommended

| Category | Drug name |
| --- | --- |
| Moderate CYP3A4/5 inhibitors | Amprenavir, atazanavir, casopitant, cimetidine, darunavir, diltiazem, fosamprenavir, lomitapide, netupitant, tofisopam, verapamil |
| Moderate CYP3A4/5 inducers | Bosentan, efavirenz, etravirine, genistein, lersivirine, modafinil, nafcillin, talviraline |
| Sensitive CYP3A4/5 substrates | Alpha-dihydroergocryptine, almorexant, alpaviroc, apixaban (doses < 2.5 mg only), atazanavir, atorvastatin, avanafil, bosutinib, brecanavir, brotizolam, budesonide, buspirone, capravirine, casopitant, darifenacin, darunavir, ebastine, eletriptan, eplerenone, felodipine, fluticasone, ivacaftor, lomitapide, lumefantrine, lurasidone, maraviroc, midazolam, perospirone, quetiapine, ridaforolimus, sildenafil, ticagrelor, tilidine, tolvaptan, triazolam, vardenafil, vicriviroc, voclosporin |
| Strong BSEP inhibitors | Bosentan, fusidate, glibenclamide, , sulindac, troglitazone (TGZ-sulfate) |
| MATE1 and OCT2 substrates | Acyclovir, amantadine, amiloride, cephalexin, cephradine, cimetidine, famotidine, fexofenadine, memantine, metformin (also a substrate for OCT1, MATE1, and MATE2K), pindolol, procainamide, ranitidine, varencicline |
| BCRP substrates | Rosuvastatin and sulfasalazine |
| Medications that carry a possible risk for QT prolongation | Alfuzosin, apomorphine, aripiprazole, atazanavir, atomoxetine, bedaquiline, clozapine, dexmedetomidine, dolasetron, eribulin, famotidine, felbamate, fingolimod, foscarnet, gatifloxacin, gemifloxacin, granisetron, iloperidone, isradipine, lithium, mirabegron, mirtazapine, moexipril, norfloxacin, ofloxacin, olanzapine, ondansetron (p.o. only at 4 mg or 8 mg), oxytocin, paliperidone, pasireotide, pipamperone, promethazine, quetiapine, ranolazine, rilpivirine, risperidone, roxithromycin, sertindole, telavancin, tetrabenazine, tizanidine, tolterodine, vardenafil, ziprasidone |

**Supplementary Table 7**: Exploratory candidate gene analysis - variants assessed by gene

| **Gene** | **Variant** |
| --- | --- |
| *ABCB1* | rs10248420, rs10276036, rs10280101, rs1045642, rs1128501, rs1128502, rs1128503, rs11983225, rs1202183, rs12720067, rs146820683, rs17064, rs17160359, rs1922242, rs2032581, rs2032582, rs2032583, rs2032588, rs2214102, rs2229107, rs2229109, rs2235012, rs2235013, rs2235015, rs2235022, rs2235033, rs2235036, rs2235039, rs2235040, rs2235044, rs2235047, rs2235048, rs2235051, rs2235067, rs2707943, rs2707944, rs28364274, rs28381801, rs28381804, rs28381867, rs28381902, rs28381914, rs28381915, rs28381966, rs28381967, rs28401781, rs28401798, rs3213619, rs3747802, rs3789243, rs3842, rs4148737, rs4148739, rs4148740, rs55852620, rs72552784, rs7787082, rs9282563, rs9282564, rs9282565 |
| *ABCG2* | rs1061018, rs13120400, rs17731538, rs17731799, rs2231135, rs2231139, rs2231142, rs2231164, rs2622604, rs2622628, rs2725256, rs2725264, rs3116439, rs3116448, rs3201997, rs41282401, rs4148157, rs45605536, rs45630471, rs58818712, rs72552713, rs72554040 |
| *CYP1A2* | rs12592480, rs1261500204, , rs12720461, rs138652540, rs143193369, rs144148965, rs149928755, rs17861157, rs183165301, rs2069514, rs2069520, rs2069521, rs2069522, rs2069526, rs2470890, rs2472304, rs28399417, rs28399419, rs28399424, rs35694136, rs35796837, rs374094758, rs3743484, rs45486893, rs4646425, rs4646427, rs55889066, rs56107638, rs56160784, rs56276455, rs71651689, rs72547511, rs72547512, rs72547513, rs72547514, rs72547515, rs72547516, rs72547517, rs758748797, rs762551 |
| *CYP2C19* | rs11188072, rs113934938, rs11568732, rs118203756, rs118203759, rs12248560, rs12571421, rs12768009, rs12769205, rs138142612, rs140278421, rs1412546060, rs144036596, rs145119820, rs1564657013, rs17878459, rs17878649, rs17878739, rs17879685, rs17879992, rs17882687, rs17884712, rs17884832, rs17885098, rs17886522, rs181297724, rs192154563, rs28399504, rs28399505, rs28399510, rs28399513, rs367543001, rs367543002, rs367543003, rs375781227, rs3758580, rs3758581, rs377184510, rs3814637, rs41291556, rs4244285, rs4417205, rs4494250, rs4917623, rs4986893, rs4986894, rs55640102, rs55752064, rs55948420, rs56337013, rs58259047, rs6413438, rs7088784, rs72552267, rs72558185, rs72558186, rs7902257, rs7916649 |
| *CYP2C9* | rs10509680, rs1057909, rs1057910, rs1057911, rs114071557, rs1237225311, rs12414460, rs1250577724, rs1274535931, rs12772884, rs12782374, rs1304490498, rs1326630788, rs141011391, rs142240658, rs146139873, rs1505, rs17847029, rs17847036, rs1799853, rs182132442, rs1934961, rs1934969, rs1934970, rs199523631, rs200965026, rs2017319, rs2256871, rs2298037, rs28371673, rs28371675, rs28371676, rs28371677, rs28371679, rs28371680, rs28371681, rs28371682, rs28371683, rs28371684, rs28371685, rs28371686, rs28371687, rs28371692, rs28371693, rs2860905, rs28969379, rs28969381, rs28969385, rs367826293, rs371055887 , rs4086116, rs4917636, rs4917639, rs4918758, rs4918798, rs56165452, rs564813580, rs57505750, rs57749228, rs61604699, rs61886768, rs61886769, rs7089580, rs71486745, rs72558187, rs72558189, rs72558190, rs72558192, rs72558193, rs749060448, rs750820937, rs754487195, rs762239445, rs764211126, rs767576260, rs769942899, rs77315016, rs774550549, rs77760615, rs781583846, rs78264032, rs78273824, rs78441737, rs868182778, rs9332092, rs9332093, rs9332094, rs9332096, rs9332098, rs9332100, rs9332101, rs9332102, rs9332104, rs9332116, rs9332119, rs9332120, rs9332127, rs9332129, rs9332130, rs9332131, rs9332172, rs9332174, rs9332197, rs9332230, rs9332232, rs9332238, rs9332239, rs9332241, rs9332242, rs9332244, rs9332245, rs988617574, rs990002631 |
| *CYP3A4* | rs1041988, rs113667357, rs11773597, rs12333983, rs12721627, rs12721629, rs12721634, rs12721636, rs1851426, rs2242480, rs2246709, rs2687116, rs2740574, rs28371759, rs28988568, rs28988569, rs3091339, rs3208361, rs3208363, rs35599367, rs3735451, rs4646437, rs4646438, rs4646440, rs4986907, rs4986908, rs4986909, rs4986910, rs4986913, rs4986914, rs4987161, rs55785340, rs55808838, rs55901263, rs55951658, rs56153749, rs56324128, rs57409622, rs59537101, rs59715127, rs67784355, rs68106838, rs6956344, rs72552794, rs72552795, rs72552798, rs72552799, rs72552800, rs72552802, rs72554603 |
| *CYP3A5* | rs10264272, rs15524, rs28365083, rs28365085, rs28365094, rs28365095, rs28371764, rs28383468, rs28383479, rs41279854, rs41279857, rs41303343, rs4646453, rs4646457, rs55817950, rs55965422, rs56244447, rs56411402, rs72552790, rs72552791 ,rs776746 |
| *FMO1* | rs10912675, rs1126692, rs12720462, rs12954, rs16864314, rs56841822, rs60639054, rs742350, rs7877 |
| *FMO3* | rs1050902, rs1050906, rs12072582, rs1736557, rs1800822, rs2066530, rs2066532, rs2066534, rs2066536, rs2266780, rs2266782, rs28363581, rs61753344, rs72549320, rs72549321, rs72549322, rs72549323, rs72549324, rs72549325, rs72549326, rs72549327, rs72549328, rs72549330, rs72549331, rs72549334, rs72549335, rs909530 |
| *NR1I2* | rs1063955, rs12721607, rs12721608, rs12721613, rs1464602, rs1464603, rs2276706, rs2276707, rs2461817, rs35761343, rs3814055, rs3842689, rs4058490, rs6785049, rs72551371, rs72551372, rs72551373, rs72551374, rs72551375, rs72551376, rs7643645, rs3814058 |
| *POR* | rs1057868, rs145782750, rs17685, rs2286824, rs6965343 |
| *SLCO1B1* | rs11045818, rs11045819, rs11045821, rs11045852, rs11045853, rs11045872, rs11045879, rs139257324, rs140790673, rs142965323, rs2291073, rs2291075, rs2306282, rs2306283, rs34671512, rs373327528, rs4149015, rs4149032, rs4149036, rs4149056, rs4149057, rs4149081, rs4363657, rs55737008, rs55901008, rs56061388, rs56101265, rs56387224, rs59113707, rs59502379, rs72559745, rs72559746, rs72559747, rs72559748, rs77271279, rs79135870 |
| *SLCO1B3* | rs11045585, rs2053098, rs3764006, rs4149117, rs4149118, rs4149143, rs72559744, rs7311358, rs7977213 |
| *SULT2A1* | rs11083907, rs11569679, rs11569680, rs11569681, rs11569685, rs17851828, rs296365 |
